# Analysis of Tp53 Codon 72 Polymorphisms, Tp53 Mutations, and HPV Infection in Cutaneous Squamous Cell Carcinomas

**DOI:** 10.1371/journal.pone.0034422

**Published:** 2012-04-24

**Authors:** Keith R. Loeb, Maryam M. Asgari, Stephen E. Hawes, Qinghua Feng, Joshua E. Stern, Mingjun Jiang, Zsolt B. Argenyi, Ethel-Michele de Villiers, Nancy B. Kiviat

**Affiliations:** 1 Divisions of Clinical Research, Fred Hutchinson Cancer Research Center, Seattle, Washington, United States of America; 2 Department of Pathology, University of Washington School of Medicine, Seattle, Washington, United States of America; 3 Department of Epidemiology, University of Washington School of Medicine, Seattle, Washington, United States of America; 4 Division of Dermatology, University of Washington School of Medicine, Seattle, Washington, United States of America; 5 Department of Medicine, University of Washington School of Medicine, Seattle, Washington, United States of America; 6 Division for the Characterization of Tumorviruses, Deutsches Krebsforschungszentrum, Heidelberg, Germany; 7 Institute of Dermatology, National Academy of Medical Sciences, Nanjing, People’s Republic of China; Ohio State University Medical Center, United States of America

## Abstract

**Background:**

Non-melanoma skin cancers are one of the most common human malignancies accounting for 2–3% of tumors in the US and represent a significant health burden. Epidemiology studies have implicated Tp53 mutations triggered by UV exposure, and human papilloma virus (HPV) infection to be significant causes of non-melanoma skin cancer. However, the relationship between Tp53 and cutaneous HPV infection is not well understood in skin cancers. In this study we assessed the association of HPV infection and Tp53 polymorphisms and mutations in lesional specimens with squamous cell carcinomas.

**Methods:**

We studied 55 cases of histologically confirmed cutaneous squamous cell carcinoma and 41 controls for the presence of HPV infection and Tp53 genotype (mutations and polymorphism).

**Results:**

We found an increased number of Tp53 mutations in the squamous cell carcinoma samples compared with perilesional or control samples. There was increased frequency of homozygous Tp53-72R polymorphism in cases with squamous cell carcinomas, while the Tp53-72P allele (Tp53-72R/P and Tp53-72P/P) was more frequent in normal control samples. Carcinoma samples positive for HPV showed a decreased frequency of Tp53 mutations compared to those without HPV infection. In addition, carcinoma samples with a Tp53-72P allele showed an increased incidence of Tp53 mutations in comparison carcinomas samples homozygous for Tp53-72R.

**Conclusions:**

These studies suggest there are two separate pathways (HPV infection and Tp53 mutation) leading to cutaneous squamous cell carcinomas stratified by the Tp53 codon-72 polymorphism. The presence of a Tp53-72P allele is protective against cutaneous squamous cell carcinoma, and carcinoma specimens with Tp53-72P are more likely to have Tp53 mutations. In contrast Tp53-72R is a significant risk factor for cutaneous squamous cell carcinoma and is frequently associated with HPV infection instead of Tp53 mutations. Heterozygosity for Tp53-72R/P is protective against squamous cell carcinomas, possibly reflecting a requirement for both HPV infection and Tp53 mutations.

## Introduction

Non-melanoma skin cancer (NMSC) is now the most common cancer among Caucasians, outnumbering the total of all other cancers combined [1]. While little mortality is associated with NMSC, these cancers constitute a major public health problem [1], [2], [3] associated with a high, and increasing financial burden [4], [5]. The pathogenesis of NMSC remains unclear. Epidemiological studies have identified many risk factors for cutaneous NMSC including UV exposure, fair complexion, older age, male sex, smoking, chronic skin ulcers and burn scars, exposure to ionizing radiation or arsenic, and immunosuppression. The importance of UV-irradiation in the development of cutaneous squamous cell carcinoma (SCC) is well established, and studies of premalignant actinic keratosis (AK) lesions and experimental skin cancers in mice suggest that a key event in the development of skin SCC is the acquisition by epidermal keratinocytes of UV-induced *Tp53* gene mutations, characteristically (C to T or CC to TT) transitions at dipyrimidine sites [6], [7], [8]. The ability of UV-irradiation to induce Tp53 mutations is important as the Tp53 gene plays a critical role in apoptosis, cell proliferation, and DNA repair. Mutations in this gene are among the most common mutations observed in human tumors, including SCC [9]. However, while inactivating Tp53 mutations are present in from 15% to over 90% of SCCs and precursors lesions [6], [10], [11], [12], [13], these mutations are also common in histologically normal skin, where they have been detected (using a variety of approaches) in 7% to 50% of samples [11], [14]. In addition, increased risk for SCC has not been a prominent feature of individuals with Li Fraumeni syndrome, which is characterized by the presence of mutations of both Tp53 alleles [15], suggesting that factors in addition to Tp53 mutations are required for the development of SCC.

Over the last 10 years there has also been increasing interest in the potential role of cutaneous human papillomavirus (HPV) in the development of SCC. However, whether certain HPVs play a role in development of skin SCC, and if so, how, is not well understood. Data supporting a role for cutaneous HPV in the development of skin SCC include a high rate of malignant transformation (i.e. development of SCC) of sun-exposed skin warts among subjects with either inherited immunosuppression (such as patients with epidermodysplasia verruciformis (EV), who lack the ability to control infection with specific cutaneous HPVs [16]), or iatrogenic immunosuppression (e.g. renal transplant recipients receiving immunosuppression [17]. Among immunocompetent subjects, detection of HPV in cutaneous SCCs has ranged from 27 to 70% [18], [19], depending on the specific PCR consensus primers employed. Several recent serologic studies using a multiplex assay have detected a higher rate in seropositivity among squamous cell carcinoma patients than among controls or basal cell carcinoma patients [20], [21], [22], [23]. In many of these studies there was a significant increase in seropositivity for β-HPV [23], [24], [25] and γ-HPV [25] in SCC patients, while other studies failed to identify the corresponding viral genomes using PCR [26]. In a previous case-control study we used a comprehensive approach for detection of HPV (employing three different PCR based protocols) to examine the relationship between the presence of specific types of HPV infection and SCC. We reported that detection of HPV DNA was high in both case lesions (54%) and perilesions (50%) and in both sun-exposed normal tissue (59%) and non-sun-exposed normal tissue (49%) from controls. However, HPV DNA from β-papillomavirus species 2 was more likely to be identified in tumors than in adjacent healthy tissue among cases (paired analysis, odds ratio =  4.0, confidence interval =  1.3–12.0) [27]. The high prevalence of β-papillomavirus species in SCC has also been noted in some [23], [28], [29], [30], [31], but not all studies [18], [32].

Laboratory based studies suggest that certain Tp53 polymorphisms may also play a role in the pathogenesis of NMSC. A common Tp53 polymorphism located at codon 72 (encoding either a proline or arginine residue), within a conserved proline rich SH3 binding domain, is known to be critical for promoting apoptosis [33], [34], and laboratory based studies have demonstrated functional and biologic differences between the two polymorphic forms [35], [36], [37], [38], [39], [40], [41]. The codon 72 polymorphism has a well-characterized geographic distribution, with the frequency of Tp53-72R increasing with geographic distance from the equator. The allelic frequency varies widely between ethnic populations, with fair skin northern European populations having a higher frequency of the Tp53-72R allele [42]. Recent epidemiologic studies have reported an association between esophagus, lung, and gastric cancers and this Tp53 codon 72 polymorphism [43], [44], [45], [46], [47], [48]. Initial studies reported that Tp53-72R was more susceptible to HPV-18 E6 mediated degradation than the Tp53-72P, and suggested that Tp53-72R is a risk factor for high risk HPV mediated cervical carcinoma [49]. However, many subsequent studies did not confirm these results [50]. Conflicting data exists on Tp53 codon 72 polymorphisms in non-melanocytic skin carcinomas (NMSCs): while some studies suggested that Tp53-72R is a risk factor for developing cutaneous SCC in immunosuppressed patients with epidermodysplasia verruciformis (EV) [51] or in post-transplant individuals [52], [53], [54], others did not [55], [56], [57], [58]. Further, recent studies have reported finding a relationship between melanoma and this polymorphism, but not in NMSC [59], [60], [61], [62].

While previous studies have examined the relationships between NMSC and HPV infection, or NMSC and UV-induced Tp53 mutations and polymorphisms, to our knowledge, none have examined all these factors in one study. In the present study, we examined the relationship between HPV infection, Tp53 mutations and Tp53 codon 72 polymorphisms in tissue biopsies of tumor and surrounding histologically normal skin from patients with NMSC, and from sun exposed and non-sun exposed tissue of individuals without NMSC. We hypothesize that several different pathways lead to the development of NMSC.

## Materials and Methods

### Study Population, Interview, and Physical Examination

The inclusion and exclusion criteria, subject recruitment, interview procedures, physical examination, and collection of specimens have been previously described [27]. Briefly, immunocompetent Caucasian men and women over the age of 55 years without a history of genetic syndromes associated with increased susceptibility to damage from UV exposure (Xeroderma pigmentosum, nevoid basal cell syndrome, albinism, epidermodysplasia verruciformis, epidermolysis bullosa dystrophica, dyskeratosis congenital), or exposure to psoralen with UV light treatment, were eligible. Digit and mucosal lip lesions were also excluded. All subjects provided informed consent according to procedures approved by the Institutional Review Boards of the University of Washington.

The present study population consisted of 55 patients (cases) and 41 controls on whom detection and sequencing of Tp53 mutations was conducted. Of the 96 study subjects, 48 (18 cases and 30 controls) were included in our previous report regarding HPV and SCC [27]. Cases were individuals with histologically confirmed cutaneous SCC who were undergoing surgical resection at the University of Washington Medical Center, the VA Puget Sound Health Care System, Group Health Cooperative, or Skin Surgery Center. Controls were individuals without a prior history of skin cancer recruited by a letter sent from their primary care providers. All control subjects had a full skin examination of their face, neck, scalp, ears, back, torso, buttocks, arms, and legs performed by a dermatologist (MMA). Those persons with lesions suspicious for SCC were deemed ineligible and referred for further evaluation. All participants completed a standardized interview to collect information pertaining to potential confounding variables including: (i) age, sex, ethnicity, income, and exposure to tobacco smoke, arsenic, hydrocarbons, or radiation; (ii) propensity to sunburn and freckle; (iii) history of pertinent dermatological conditions, including actinic keratosis, burn scars, ulcers, and warts; and (iv) lifetime history of sun exposure, including a history of tanning bed use, and number of blistering sunburns.

### Collection of Skin Specimens

For cases, a portion of the residual SCC along with adjacent clinically uninvolved perilesional skin was collected from each subject. For controls, punch biopsies of clinically normal skin were obtained from a sun-exposed (pre-auricular) site as well as from a sun-protected site from the upper inner arm as previously described. One third of the biopsy was placed in 10% neutral buffered formalin for histopathology, another third placed into specimen transport media (STM, Qiagen), and the final portion placed into ethanol.

### Histopathology and Detection of HPV Infection

Routine paraffin-embedded tissue was prepared from the biopsy specimens and then sectioned and stained with hematoxylin and eosin to confirm the diagnosis. HPV was detected by PCR and typed as previously described using three protocols to assure detection of all HPV types [27].

### Detection and Sequencing of Tp53 Mutations and Polymorphism

Since our goal was to identify major Tp53 mutations that are likely involved in the pathogenesis of neoplasia, we focused on identifying clonal mutations (most abundant in neoplastic or pre neoplastic tissue), rather than sub-clonal mutations (present at lower frequencies). To this end we directly sequenced coding sequence (cDNA) amplified from tissue specimens without employing a clonal enrichment steps [63]. The mRNA was extracted from freshly frozen biopsies using TRIzol reagent (Invitrogen) according to the manufacturer protocol. Briefly, the frozen tissue was homogenized in TRIzol reagent and incubated at RT for 5 minutes. The homogenate was centrifuged to pellet polysaccharides and lipids. Chloroform was then added to the supernatant, mixed and centrifuged. The RNA in the upper aqueous phase was isolated by ethanol precipitation. The mRNA template was transcribed into cDNA with MLV reverse transcriptase, using p53 specific primers. Nested PCR reactions were carried out to amplify an 873 bp fragment from exon 4 to exon 8, spanning the region that encodes most of the allelic polymorphisms and reported mutations (Table S1). Two negative controls (reaction mix without cellular DNA) were included in each set of reactions to monitor potential contamination. PCR products were isolated by anion exchange using the QIAEX II gel extraction kit (Qiagen Inc., Chatsworth, CA) and 50–100 ng of the PCR product was sequenced using ABI PRISM™ Dye Terminator Cycle Sequencing kit (Perkin-Elmer Cetus) on an Applied Biosystem Model 373A DNA sequencing system (Applied Biosystems Inc.). Each sequence was determined bidirectionally using the nested primers (Table S1). Resulting sequences were analyzed with Sequencher™ (Gene Codes Corp., Ann Arbor, MI) and compared to the wild type Tp53 sequence.

Tp53 sequence was evaluated for the presence of either non-synonymous (altering the amino acid sequence), synonymous (no change in amino acid sequence) mutations and UV damage signature (transitions at dipyrimidine sites, C>T or tandem CC>TT) [6]. Determination of the common Tp53 polymorphism alleles at codon 72 was from direct sequencing of the cDNA amplicon. Tp53 polymorphism at codon 72 present in lesional (i.e. tumor) and perilesional (i.e. normal) tissues in cases, and in sun exposed and non sun exposed tissue in the controls was conserved with discrepancy in only 3 of the 55 paired lesional/perilesional samples, possibly due to loss of heterozygosity (LOH) or preferential allelic expression. Hotspot mutations were defined as those categorized as signatures of UV damage and occurring frequently in NMSCs [6], [8], [12], they included Arg248Trp, Arg196Stop, and His179Stop.

### Statistical Methods

Differences between cases and controls were assessed using chi-square analyses to compare categorical variables and Student’s t-test to compare continuous measures. Associations with ordinal factors, such as propensity to sunburn, were evaluated with Mantel-Haenszel tests for trends. Fisher’s exact tests were utilized when cell frequencies were less than 5. To evaluate differences in categorical measures by site of the skin tissue, paired samples from within a subject were compared using McNemar’s Test.

A two-sided 0.05 level test determined statistical significance for all analyses. All analyses were conducted using SAS 9.1 (SAS Institute Inc., Cary, NC).

## Results

The characteristics of the study population including 55 subjects with and 41 without cutaneous SCC are presented in Table 1. Overall, the patients were primarily elderly immunocompetent males. The cases and controls were similar regarding gender, history of smoking, and family history of skin cancer. However, the case population was slightly older and had an increased propensity to sunburn compared to the control population.

**Table 1:** Demographic and Skin Characteristics of Cases with Squamous Cell Carcinoma and Controls.

	Cases with SCC (n = 55)	Controls (n = 41)	P-value
**Age (mean years ± sd)**	74.5	69.9	0.02
**Age**			0.08
** <70**	17 (31%)	21 (51%)	
** 70–79**	21 (38%)	11 (27%)	
** ≥80**	17 (31%)	9 (22%)	
**Female Gender**	2 (4%)	5 (12%)	0.13
**Ever Smoker**	42 (76%)	31 (76%)	0.93
**Family Hx of Skin Cancer**	10 (20%)	5 (13%)	0.39
**Propensity to Sunburn**	48 (87%)	25 (61%)*	0.003
**Propensity to Sunburn**			0.001
** Burns, blisters**	14 (25%)	5 (12%)*	
** Burns, no blisters**	18 (33%)	4 (10%)	
** Mild burn, tans**	16 (29%)	16 (39%)	
** No burn, tans**	4 (7%)	13 (32%)	
** No burn, no tan**	3 (5%)	3 (7%)	

### Frequency of Tp53 Mutations in Lesional, Perilesional and Control Samples

Among cases, the frequency of any Tp53 mutation was significantly increased in the lesional samples (33%) as compared to perilesional samples (16%). However, Tp53 mutations were rare in both sun exposed (2%) and sun protected (5%) control tissues (Table 2). Identification of more than one type of Tp53 mutation was noted in 7% of lesional samples, 2% of perilesional samples, 5% of sun protected tissue from controls and 0% from sun-exposed areas from controls.

**Table 2:** Tp53 mutation and HPV DNA detection in skin lesions, perilesions and non-lesional control samples from subjects with and without SCC.

	SCC subjects		Control subjects		P1 (les vs. peri)		P2 (les vs. sun)		P3 (peri vs. sun)
	Lesional tissue (n = 55)	Perilesion tissue (n = 55)	Non-lesional sun-exposed tissue (n = 41)	Non-lesional sun-protected tissue (n = 41)					
**Tp53 Mutations**									
** Any**	18 (33%)	9 (16%)*	1 (2%)**	2 (5%)	0.03	<0.001		0.04	
** Single**	14 (25%)	8 (15%)	1 (2%)	0 (0%)					
** Multiple**	4 (7%)	1 (2%)	0 (0%)	2 (5%)					
**Non-synonymous coding mutations**									
** Any**	17 (31%)	8 (15%)*	0 (0%)**	2 (5%)	0.03	<0.001		0.01	
** Single**	14 (25%)	7 (13%)	0 (0%)	1 (2%)					
** Multiple**	3 (5%)	1 (2%)	0 (0%)	1 (2%)					
**UV-damage C→T or CC→TT transition mutations**									
** Any**	13 (24%)	7 (13%)	0 (0%)**	2 (5%)	0.11	<0.001		0.02	
** Single**	12 (22%)	6 (11%)	0 (0%)	1 (2%)					
** Multiple**	1 (2%)	1 (2%)	0 (0%)	1 (2%)					
**“Hotspot” Mutations** ^									
** Any**	11 (20%)	4 (7%)	0 (0%)**	0 (0%)	0.05	0.002		0.13	
** Single**	10 (18%)	4 (7%)	0 (0%)	0 (0%)					
** Multiple**	1 (2%)	0 (0%)	0 (0%)	0 (0%)					
**HPV DNA**	35/53 (66%)	29/53 (55%)	24/40 (60%)	21/41 (51%)	0.13	0.55		0.61	
**Beta 2 HPV Species**	4/20 (20%)	4/20 (20%)	17/40 (43%)	14/41 (34%)	1.00	0.15		0.15	

* p-value<0.05 for cases compared to perilesions (McNemar’s Test).

** p-value<0.05 for cases compared to sun-exposed controls (Chi-Square test).

^ Hotspots include Arg248Trp, Arg196Stop, and His179Stop.

Characterization of the specific type of Tp53 mutations showed that 17 of 18 lesional tissues with Tp53 mutations were non-synonymous mutations (resulting in amino acid changes), and 13 of these had mutations with a UV damage signature (i.e., C>T or tandem CC>TT transitions at dipyrimidine sites). 8 of 9 Tp53 mutations from perilesional tissue were non-synonymous, with 7 exhibiting a UV damage signature. The single Tp53 mutation identified in the sun-exposed control was synonymous (no amino acid change), while the two mutations identified in the sun-protected controls were non-synonymous mutations with a UV damage signature. Among tumor tissues with Tp53 mutations, 11 of 18 (61%) were previously reported “hotspot” mutations, while among perilesional tissues 4 of 9 (44%) were “hotspot” mutations. Surprisingly only 1 of 5 (20%) of the lesional and perilesional tissues showed concordant Tp53 mutations.

### Tp53 Pro-72 Polymorphism in Cases and Controls

Since, as mentioned above some [61], but not all [50], [58], previous studies have demonstrated an association between the polymorphism at codon Tp53-72 and the development of cancer, we examined the distribution of the common Tp53 codon-72 polymorphism among the case and controls (Table 3). The presence of at least one Tp53-72P allele (Pro/Pro or Pro/Arg) was significantly higher in the control population (59%) when compared to the SCC population (36%). The presence of at least one Tp53-72R did not differ between SCC (93%) and control (88%) populations.

**Table 3:** Codon-72 Polymorphism in Cases and Controls.

	Cases (lesions) (n = 55)	Controls (sun-exposed) (n = 41)	P-value
**Polymorphism**			0.10
** Pro/Pro**	4 (7%)	5 (12%)	
** Pro/Arg**	16 (29%)	19 (46%)	
** Arg/Arg**	35 (64%)	17 (41%)	
**Any Proline (Pro/Pro or Pro/Arg)**	20 (36%)	24 (59%)	0.03

### Detection of HPV DNA in Lesional, Perilesional and Control Samples

In the present study, 66% of the lesional samples, 55% of the perilesional samples, 60% of the sun-exposed control samples, and 51% of the sun-protected control samples had detectable HPV DNA. Although we previously reported that HPV β-papillomavirus species was differentially detected in lesional (tumor) as compared to perilesional tissue in the larger sample set, there was no difference between lesional, perilesional and control tissues with respect to the specific types of HPV present in this smaller sample set. Interestingly, detection of HPV was inversely associated with the presence of Tp53 mutations among lesion tissues (Table 4). While Tp53 mutations were present in 50% of the HPV negative cancers, they were only detected in 23% of HPV positive cancers (p = 0.045). Among perilesional tissues, Tp53 mutations were at similar frequencies in HPV negative samples (4/24, 17%) and in HPV positive samples (6/29, 21%) (data not shown).

**Table 4:** Tp53 mutations and Detection of HPV DNA in Lesional Samples of Cases with SCC.

Type of Tp53 Mutation	HPV DNA Positive	HPV DNA Negative	P-value
**Any mutation(s)**	8/35 (23%)	9/18 (50%)*	0.045
**Non-synonymous coding mutation(s)**	8/35 (23%)	9/18 (50%)*	0.045
**UV-damage C→T or CC→TT transition mutation(s)**	5/35 (14%)	7/18 (39%)*	0.08
**“Hotspot” mutation(s)**	5/35 (14%)	6/18 (33%)	0.15
** Arg248Trp**	3/35 (9%)	1/18 (6%)	1.00
** Arg196Stop**	1/35 (3%)	1/18 (6%)	1.00
** His179Stop**	1/35 (3%)	3/18 (17%)	0.11

### Relation between Codon 72 Polymorphism, HPV Infection and Tp53 Mutations in Cutaneous Squamous Cell Carcinoma

Given the above findings, we asked whether the role of Tp53 mutations and of HPV infection in the pathogenesis of SCC might vary with a specific polymorphism at Tp53-codon 72 (Table 5). Among lesion tissues, the detection of HPV was not statistically different among patients with (12/20, 60%) or without (23/33, 70%) the Tp53-72P polymorphism. In contrast, Tp53 mutations were more likely to be detected from individuals having a Tp53-72P allele (11/20, 55%) as compared to those without (7/35, 20%) (p = 0.0078). Similarly, in perilesional tissues, Tp53 mutations were detected in 7 of 19 (37%) patients who had a Tp53-72P polymorphism as compared to 2 of 36 (6%) without Tp53-72P. However, HPV DNA was detected at similar frequencies from those with (11/19, 58%) or without a Tp53-72P polymorphism (18/34, 53%) (Table 6). No significant difference in the frequency of Tp53 mutations was detected in control individuals with or without the Tp53-72P polymorphism.

**Table 5:** Relationship between Codon-72 polymorphisms, HPV, and Tp53 Mutations in Lesional Samples.

	Proline-72 Positive (CC or CG)	Proline-72 Negative (GG)	P-value
**HPV positive**	12/20 (60%)	23/33 (70%)	0.47
**Any Tp53 mutation(s)**	11/20 (55%)	7/35 (20%)	0.008
**Non-synonymous coding mutation(s)**	11/20 (58%)	6/35 (17%)	0.004
**UV-damage C→T or CC→TT transition mutation(s)**	8/20 (40%)	5/35 (14%)	0.048
**“Hotspot” mutation(s)**	7/20 (35%)	4/35 (11%)	0.08
** Arg248Trp**	2/20 (10%)	2/35 (6%)	0.62
** Arg196Stop**	2/20 (10%)	0/35 (0%)	0.13
** His179Stop**	3/20 (15%)	1/35 (3%)	0.13

**Table 6:** Relationship between Codon-72 polymorphisms, HPV, and Tp53 Mutations in Perilesional Samples.

	Proline-72 Positive (Pro/Pro or Pro/Arg)	Proline-72 Negative (Arg/Arg)	P-value
**HPV Positive**	11/19 (58%)	18/34 (53%)	0.73
**Any Tp53 mutation(s)**	7/19 (37%)	2/36 (6%)	0.005
**Non-synonymous coding mutation(s)**	7/19 (37%)	1/36 (3%)	0.002
**UV-damage C→T or CC→TT transition mutation(s)**	6/19 (32%)	1/36 (3%)	0.005
**“Hotspot” mutation(s)**	4/19 (21%)	0/36 (0%)	0.01

Table 7 summarizes the relationship between the presence of HPV DNA, Tp53 mutations and Tp53-72P status. Most cancers (44/53, 86%) contained either HPV or Tp53 mutations. While there was increased detection of Tp53 mutations in SCC arising among those with (11/20, 55%), as compared to those without (6/33, 18%) a Tp53-72P polymorphism (p = 0.005), detection of HPV was similar among patients with (12/20, 60%) or without (23/33, 70%) the Tp53-72P polymorphism. However, in patients without Tp53 mutations, HPV DNA was somewhat less likely detected in patients with (7/20, 35%), as compared to those without (20/33, 61%) the codon-72P polymorphism (p = 0.07). Samples from patients heterozygous for codon-72R/P were somewhat more likely to contain both HPV DNA and Tp53 mutations (5/16, 31%) and were somewhat less likely to be negative for HPV DNA and Tp53 mutations (1/16, 6%; p = 0.07).

**Table 7:** HPV DNA and Tp53 Mutations Vary by Codon-72 Polymorphisms in Skin Cancer.

	Proline 72 positive (Pro/Pro)	Proline 72 positive (Pro/Arg)	Proline 72 negative (Arg/Arg)
**Neither**	1/4 (25%)	1/16 (6%)	7/33 (21%)
**HPV, no Mutations**	1/4 (25%)	6/16 (38%)	20/33 (61%)
**Mutations, No HPV**	2/4 (50%)	4/16 (25%)	3/33 (9%)
**HPV and Mutations**	0/4 (0%)	5/16 (31%)	3/33 (9%)

## Discussion

Non-melanoma skin cancers are frequent tumors that arise from a complex interaction between environmental, immunologic and genetic factors [64], [65]. The multifactorial nature of these cancers has led to much controversy and speculation with regard to etiologic mechanisms. The aim of this study was to better define the causal association of HPV infection and the tumor suppressor gene Tp53 (polymorphism and mutations) in cutaneous squamous cell carcinomas. We found a significant increase in the frequency of Tp53 mutations in carcinoma and perilesional samples compared to controls, with the majority of these mutations being non-synonymous changes. In contrast, the majority of samples were positive for HPV infection, determined by the extremely sensitive PCR assay. There was only a moderate increase in the incidence of HPV infection in the carcinomas above the high steady state level detected in perilesional or control samples. There was, however, an inverse correlation between the presence of HPV and Tp53 mutations in the carcinoma specimens; 50% of HPV negative carcinomas had Tp53 mutations while only 23% of the HPV positive specimens had Tp53 mutations. These results suggest that two distinct molecular pathways either HPV infection or Tp53 mutations may drive carcinogenesis. We further found that the frequency of Tp53 mutations and HPV infection correlated with the identity of the common codon-72 Tp53 polymorphism. Homozygosity for the Tp53-72R allele was a significant risk factor for the development of squamous cell carcinomas. Among patients with SCC, the presence of Tp53-72P allele was associated with Tp53 mutations, while the lack of Tp53-72P allele was associated with HPV infection. Patients heterozygous for Tp53-72R/P are associated with an increased frequency of both Tp53 mutations and HPV infections.

### Tp53 Mutations and Cancer

As expected there was an increase in the frequency of Tp53 mutations in the tumors compared with either the perilesional or control specimens. Most of the Tp53 mutations present in the cancers were non-synonymous changes and many were previously characterized “hotspots” that showed signatures of UV damage suggesting exposure to sunlight is a primary environmental insult causing the mutations [6], [8], [12]. The most frequently identified mutations were Arg248Trp, His179Tyr and Arg196Stop. Tp53-Arg248Trp is a common UV damage-associated mutation that arises from CC>TT transition at a dipyrimidine site that spans two adjacent codons mutations. Arg248Trp (R248W) disrupts DNA binding and gain of function effect in Tp53 null cells, promoting tumorigenesis in nude mice and growth in soft agar [66]. Tp53-His179Tyr maps to the conserved L2 loop and has been shown to prevent zinc binding resulting in a non-functional protein [67], [68], [69]. Tp53-Arg196Stop is a nonsense mutation resulting in premature termination and the generation of an inactive protein [68], [69]. In contrast, most of the Tp53 mutations identified in the control samples were silent synonymous changes that do not alter the amino acid residues. The presence of non-synonymous mutations in the carcinoma suggests that the Tp53 mutations provide the cell with a selective growth advantage by inhibiting apoptosis and promoting genetic instability. On the other hand, Tp53 mutations in the control samples were random passenger mutations that did not confer a selective growth advantage. It is possible that some of the silent Tp53 passenger mutations in the normal control skin may represent rare heritable variants since normal non-skin tissue was not available for comparison.

For this study we were interested in identifying the most prevalent p53 mutations that might represent driver mutations promoting SCC. Therefore we choose to use direct cDNA sequencing without a clonal enrichment step such as denaturing high-performance liquid chromatography (DHPLC). This procedure allows for greater specificity in identifying clinically relevant mutations that promote carcinogenesis. However this approach does not detect every p53 mutation present in the sample since small subclonal mutations would not be identified without a clonal enrichment step. In addition, our sequencing strategy targeted exon 4 to exon 8, spanning the region that encodes most of the allelic polymorphisms and reported mutations. P53 mutations occurring outside of this region would not be detected in this study. Predominant mutations in Tp53 were readily identified in tumors representing a clonal population of cells sharing identical Tp53 mutations. Presumably, p53 sequences amplified from control and perilesional samples were heterogeneous and contained subclonal Tp53 mutations amid a background of wild-type Tp53 sequences; minor Tp53 mutations were not detected by direct sequencing. The frequency of Tp53 mutations detected in the perilesional and control specimens were lower than that reported in other studies focusing on squamous cell carcinomas that employed a clonal enrichment procedure [70].

In a recent study Durinck et al. determined the temporal sequence of acquired genetic abnormalities during tumor progression by using a combination of DNA sequence and copy number variations [71]. They conclude that the development of most tumor-associated mutations in squamous cell carcinoma are gated by the elimination of the wildtype p53 allele (LOH). Unfortunately the methodology used in our studies (cDNA sequencing) does not allow us to accurately determine ploidy. Only the mutated template was visible in the vast majority of the mutations identified in the lesion and perilesions specimens and thus is either homozygous (LOH) or reflects the preferential expression of the mutant allele.

Of the 56 paired samples (lesional and perilesional) only 5 had Tp53 mutations identified in both tissues and only 1 (20%) shared the same mutation. The lack of tumor-specific p53 mutations in the adjacent perilesional is probably due to sampling bias and the limited sensitivity of direct cDNA sequencing. Ren et al. found that benign clonal keratinocyte patches with p53 mutations showed no genetic correlation to adjacent squamous cell carcinomas. These authors suggest the p53 expressing keratinocyte patches result form clonal expansion of cells with p53 mutations that may be permissive for a subsequent event driving carcinogenesis. In the vast majority of cases these p53 mutations do not represent precursor mutations present in the adjacent carcinomas [11], [72]. Alternatively, the causal Tp53 mutations may be late events in carcinogenesis and may not be present in the adjacent precursor lesions. Additional studies using more sensitive methodology are required to identify the presence of tumor specific p53 mutations in adjacent perilesional biopsies.

### HPV Infection and Cancer

Although direct causal association between HPV and cervical carcinoma has been well established [73], [74], [75], the association between HPV and cutaneous squamous cell carcinomas is less well defined. In our previous studies the high prevalence and widespread distribution of HPV species made it statistically difficult to associate the causal link between selective HPV genotypes and squamous cell carcinomas. The prevalence of HPV DNA in the tumors was not significantly elevated above the high level present in the control samples [27]. In addition there was no differential detection of HPV DNA across various HPV types [27]. However, we and others [76] found that HPV DNA from β-papillomavirus species 2 was more likely to be present in tumors than adjacent healthy tissue, suggesting that certain HPV types may be involved in the progression of cutaneous SCCs. The current study supports the results of the previous study in that there was no significant increase in HPV DNA in cases (66%) compared to either perilesional (55%) or control samples (60%). These results suggest that in most cases HPV infection alone is not sufficient to promote carcinogenesis; additional factors such as perhaps immunosuppression or additional genetic modifications are required. While some serological studies have shown that patients with correlated detection of HPV antibodies and β-papillomavirus have increased risk of developing squamous cell carcinoma [20], other studies have shown a lack of correlation between the presence of HPV DNA and antibodies suggesting the antibody may have arisen from a remote site or prior infection [26]. In addition, a recent study employing transcriptome sequencing failed to detect HPV gene expression in squamous cell carcinomas suggesting that papillomavirus expression is not a factor in maintenance of cutaneous SCC [77]. In our study HPV detection was based on an extremely sensitive PCR assay that did not provide information on viral load, transcriptional activity, or viral integration that could help to determine the role of HPV in cutaneous carcinogenesis. It is also likely that specific cutaneous HPV types are more pathogenic than others; however, a larger clinical study is required to identify the more pathogenic types.

### Incidence of HPV Infection and Tp53 Mutations

We hypothesized that HPV infection and Tp53 mutations would be increased in carcinoma samples with a positive and cooperative association in promoting carcinomas. Instead, we found an inverse association; HPV positive tumors had a lower incidence of Tp53 mutations (23%) in comparison to HPV negative tumors (50%). This inverse association was not present in perilesional or control samples suggesting that this relationship pertains to the induction of tumors. The simplest explanation for these results is that two potential separate pathways either dependent on HPV infection or on Tp53 mutation lead to cutaneous squamous cell carcinoma. HPV infection may decrease the incidence or necessity of Tp53 mutations, and Tp53 mutations may inhibit HPV infection or the proliferation of HPV infected cells. A similar inverse correlation has been reported in oropharyngeal squamous cell carcinomas [78] and head and neck carcinomas [79], [80], [81] in which HPV positive tumors were associated with wild type TP53 and HPV negative tumors had a higher frequency of Tp53 mutations. In contrast, recent *in vitro* studies have shown that expression of wt Tp53 can inhibit HPV replication by blocking the expression, and promoting the degradation of HPV20 E6 [82], while the common hot spot mutation R248W confers a protective effect on the HPV20 E6 protein to promote viral replication [83].

Although the E6 and E7 proteins encoded by cutaneous HPVs do not bind or target Tp53 or RB protein for degradation [84], the E6 protein encoded by β-papillomavirus does bind and inactivate the pro-apoptotic protein BAK [85]. The elimination of BAK inhibits apoptosis, resulting in a partial downstream block of Tp53 function, which may abrogate the need for Tp53 inactivation by mutation or degradation [86].

### Tp53-72R/P Polymorphism Association with Squamous Cell Carcinoma

By stratifying patients based on the status of the common Tp53 polymorphism at codon-72(R/P) we identified several additional statistically significant associations that lead to important mechanistic predictions. The lack of Tp53-72P (Tp53-72R/R) was a significant risk factor for SCC in the study population of elderly white male patients. Tumors without Tp53-72P (Tp53-72R/R) had fewer Tp53 mutations with a small non-significant increase in HPV DNA. These results suggest that patients homozygous for Tp53-72R are more likely to develop cutaneous squamous cell carcinoma with HPV infection and without Tp53 mutations. These results resemble those reported for SCC arising in immunosuppressed patients. It is well documented that there is an increased incidence of skin cancers in selective immunosuppressed patient populations (solid organ transplant and epidermodysplasia verruciformis (EV)) attributed to potential infectious or immune-modulatory factors [87]. Persons afflicted with EV, an inherited disorder of cell immunity, are unable to control cutaneous HPV infection leading to flat, atypical warts that eventually undergo malignant transformation [87], [88]. Solid organ transplant patients also develop a large number of warts that carry a high risk of malignant transformation [18]. Studies focusing on cutaneous malignancies arising in these immunosuppressed patient populations have reported the lack of Tp53-72P to be an additional risk factor for developing squamous cell carcinoma [89], [90]. In our study population of elderly white males presumably with weakened immune systems, the lack of Tp53-72P is a risk factor for developing SCC frequently associated with HPV DNA. These results are in accord with recent studies in head and neck carcinomas that showed the risk of HPV mediated tumorigenesis increased with the numbers of Tp53-72R alleles [79].

The codon-72 polymorphism is located in a highly conserved proline rich domain involved in DNA binding, and several studies show clear functional differences between the two isoforms. Expression of the Tp53-72R in p53-null cells induced enhanced apoptosis possibly related to increased localization of the arginine variant to the mitochondria compared to the proline variant [34]. In addition, the Tp53-72R isoform binds more strongly to the proly-isomerase PIN1 resulting in increased p53 acetylation and dissociation from the apoptosis inhibitor iASPP than the Tp53-72P form [91], supporting the view that Tp53-R72 is a more potent inducer of apoptosis than Tp53-P72. The enhanced tissue specific apoptotic potential of the Tp53-72R variant was recently confirmed in mouse models for the codon-72 Tp53 polymorphism [37], [40]. Cell culture based studies particularly in dermal fibroblasts derived from centurions [92] have shown that expression of the Tp53-72P variant results in growth arrest and senescence due to increased ability to transactivate p21/waf1 [41]. Cell expressing the Tp53-72P variant also are more efficient at specifically activating several Trp53-dependent DNA-repair target genes and are associated with a higher DNA repair capacity than cells expressing the Tp53-72R allele [93]. The current consensus is that Tp53-72R is more effective at inducing apoptosis and protecting stressed cells from neoplastic development while Tp53-72P is more effective at inducing cell cycle arrest and senescence.

Similar to the cell cycle studies the association of the common Tp53 polymorphisms at codon-72 with cancer risk has been extensively studied for multiple cancers with mixed and inconsistent results. There are over 230 studies in the NIH genetic association database evaluating the effect of the codon-72 polymorphism on susceptibility to a wide variety of cancers with many reporting ‘statistically significant’ associations [50]. Of the studies that report a significant tumor association, the majority claim Tp53-72P to be a risk factor for developing malignancy [94], [95] or to be associated with higher stage of disease or poor prognostic outcome [96]. A smaller subset of studies reported Tp53-72R to be a risk factor for the development of cancer in immunocompetent [97] and immunosuppressed patients [98]. In contrast, many other studies have failed to identify a significant association between the polymorphism and cancer [50]. The variability in results is attributed to the multifactorial etiology of cancer and study participant characteristics since the Tp53 polymorphism varies widely among ethnic groups.

### Tp53-72R and HPV Associated Carcinomas

Among the carcinomas without detectable Tp53 mutations there was a non- significant trend toward an increased frequency of HPV infection in tumors lacking Tp53-72P (Table 7). This trend was not present when tumors with Tp53 mutation were included and suggests that the effect of HPV infection in promoting carcinomas is greatest in cells without Tp53-72P isoforms or Tp53 mutations. This suggests that the role of HPV in promoting carcinomas may be through inhibiting the Tp53 pathway and that cells expressing mutant Tp53 and Tp53-72P may not be less susceptible to HPV induced carcinogenesis. The selective role of HPV in homozygous Tp53-72R/R carcinomas may be related to the increased and altered capacity of that polymorphic isoform to induce apoptosis by direct mitochondrial binding [34] that may be partially inhibited by HPV infection [99]. The association of HPV with cells homozygous for Tp53-72R/R is probably not due to permissive replication of the virus in these cells since we showed there was no significant increase in HPV detected in homozygous Tp53-72R/R perilesional and control specimens.

Our results show that tumors expressing the Tp53-72P polymorphism showed an increased frequency of Tp53 mutations (55%) in comparison to tumors without Tp53-72P (20%) consistent with studies in lung carcinoma [100] [47]. These results may reflect a selective growth advantage imparted to Tp53-72P cells expressing mutant forms of Tp53 or may reflect a mutator phenotype resulting in enhanced genome wide mutations. Studies have shown that the expression of the Tp53-72P allele in Glioblastoma [101], ovarian carcinoma [102], and head and neck carcinoma [103] is associated with more aggressive disease and a shorter survival.

In a recent study, Guan et al. showed that ARID1a, a tumor suppressor gene involved in chromatin remodeling and frequently mutated in a wide variety of tumors binds to p53 [104]. The p53/ARID1a complex is required to direct the transcription of several p53 inducible genes. In addition, they show a mutually exclusive pattern of Tp53 and ARID1a mutations, with wild-type p53 tumors containing mutant ARID1a and mutant p53 tumors containing wildtype ARID1a. It will be important to investigate the status of ARID1a in squamous cell carcinomas and to determine the relationship with p53 mutations, polymorphisms and HPV infection.

In conclusion, our studies demonstrate a clear association between Tp53 mutations and squamous cell carcinomas. The association with HPV infection is less well defined partly due to the high incidence of HPV infection in normal and tumor biopsies. It is possible that specific HPV types are causally linked with cutaneous squamous cell carcinomas and were not identified in this study since all HPV infections were classified together. Studies monitoring other aspects of HPV infection including viral load, integration and expression may better define a causal link with cutaneous squamous cell carcinoma. Importantly we found that the expression of the common Tp53 polymorphism Tp53-72P is protective against the development of cutaneous squamous cell carcinomas arising in elderly white males. In addition, we found a significant inverse correlation between Tp53 mutations and HPV DNA in the tumors stratified for Tp53-72 polymorphism suggesting two separate mechanisms of carcinogenesis one dependent on Tp53 mutations and the other dependent on HPV infection.

## Supporting Information

Table S1 **Primers for Tp53 analysis.** The Tp53 mRNA template was transcribed into cDNA with MLV reverse transcriptase, using the listed cDNA amplification primers (1) to generate a 1142 bp product. Nested PCR reactions were carried out to amplify an 873 bp fragment from exon 4 to exon 8 using the nested PCR amplification primers (2). Both strands of the amplified cDNA were then sequenced using the specific sequencing primers (3).
(DOCX)
