# Supplementary material for: Analysis of Tp53 Codon 72 Polymorphisms, Tp53 Mutations, and HPV Infection in Cutaneous Squamous Cell Carcinomas
Source: PLoS One. 2012 Apr 24;7(4):e34422. doi: 10.1371/journal.pone.0034422 (PMC3335843; doi:10.1371/journal.pone.0034422)
Supplement: Table S1 — Primers for Tp53 analysis. The Tp53 mRNA template was transcribed into cDNA with MLV reverse transcriptase, using the listed cDNA amplification primers (1) to generate a 1142 bp product. Nested PCR reactions were carried out to amplify an 873 bp fragment from exon 4 to exon 8 using the nested PCR amplification primers (2). Both strands of the amplified cDNA were then sequenced using the specific sequencing primers (3). (DOCX) [file pone.0034422.s001.docx]

**Table S1. Primers for Tp53 analysis**

1. cDNA amplification (1142 bp product)

P53-F3 CCCCTCTGAGTCAGGAAACA

P53-R3 AGACAGAAGGGCCTGACTCA

2. Nested PCR amplification (989 bp product)

P53-F2 TGTCCCCGGACGATATTGAAC

P53-R2 CACCTGAAGTCCAAAAAGGGT

3. Sequencing primers (807 bp product)

P53seqr tccatccagtggtttcttc

P53seqf gacccaggtccagatgaagc
